# Molecular Targeted Therapy in Myelodysplastic Syndromes: New Options for Tailored Treatments

**DOI:** 10.3390/cancers13040784

**Published:** 2021-02-13

**Authors:** Simona Pagliuca, Carmelo Gurnari, Valeria Visconte

**Affiliations:** 1 Department of Translational Hematology and Oncology Research, Taussig Cancer Institute, Cleveland Clinic, Cleveland, OH 44195, USA; paglius@ccf.org (S.P.); gurnarc@ccf.org (C.G.); 2 Hematology, Oncogenesis and Biotherapies, University of Paris, 75013 Paris, France; 3 Department of Biomedicine and Prevention, Molecular Medicine and Applied Biotechnology, University of Rome Tor Vergata, 00133 Rome, Italy

**Keywords:** MDS, HMA failure, targeted therapies

## Abstract

**Simple Summary:**

Myelodysplastic syndromes (MDS) are a group of diseases in which bone marrow stem cells acquire genetic alterations and can initiate leukemia, blocking the production of mature blood cells. It is of crucial importance to identify those genetic abnormalities because some of them can be the targeted. To date only very few drugs are approved for patients manifesting this group of disorders and there is an urgent need to develop new effective therapies. This review gives an overview of the genetic of MDS and the therapeutic options available and in clinical experimentation.

**Abstract:**

Myelodysplastic syndromes (MDS) are a heterogeneous group of clonal hematopoietic disorders characterized by ineffective hematopoiesis, progressive cytopenias and increased risk of transformation to acute myeloid leukemia. The improved understanding of the underlying biology and genetics of MDS has led to better disease and risk classification, paving the way for novel therapeutic opportunities. Indeed, we now have a vast pipeline of targeted agents under pre-clinical and clinical development, potentially able to modify the natural history of the diverse disease spectrum of MDS. Here, we review the latest therapeutic approaches (investigational and approved agents) for MDS treatment. A deep insight will be given to molecularly targeted therapies by reviewing new agents for individualized precision medicine.

## 1. Introduction

Myelodysplastic syndromes (MDS) constitute the paradigm of clonal hematopoietic disorders, characterized by abnormal bone marrow cell morphology, peripheral cytopenias and an increased risk of evolution to acute myeloid leukemia (AML) [1,2]. Patient disease course, incidence of progression and survival outcomes are highly variable, based on genomic features, risk stratification, symptom burden and comorbidities.

A major hallmark of MDS pathophysiology is the acquisition of genetic aberrations in hematopoietic stem cells and progenitors leading to abnormal cell proliferation and loss of differentiation potential. Since the last two decades many progresses have been made in dissecting the molecular pathogenesis of MDS.

The extensive sequencing of the genomes of large cohorts of MDS patients during the last 10 years has exponentially increased the spectrum of possibilities to identify patterns of somatic mutations shaping the clonal architecture of these disorders, and contributing to distinct disease phenotypes, possibly impacting patient outcomes [3,4,5]. A number of genes (e.g., splicing factor 3b subunit 1 (*SF3B1*) and ten-eleven translocation- 2 (*TET2*)) have been identified as recurrently mutated in primary MDS patients, playing a role in the pathogenesis of the disease as driver mutations. Besides these, many other somatic hits (passenger mutations) have been found acquired in individual cells during life, albeit with a different pathogenic potential [4,6].

Most of the frequent somatic driver hits occur in genes clustering in functional groups pertaining to: DNA methylation, chromatin modification, signal transduction, cohesin complex, RNA splicing, transcription and DNA repair pathways (Table 1) [4,5,7,8].

The level of complexity increases in the case of patients with germline mutational configurations associated with inherited bone marrow failure (BMF) disorders, (including dyskeratosis congenita, Fanconi anemia, Down syndrome, RASopathies, severe congenital neutropenia, Shwachman–Diamond syndrome, and xeroderma pigmentosum), or less classical germline syndromes predisposing to myeloid neoplasms such as those associated with inherited GATA binding protein 2 (*GATA2*), Sterile Alpha Motif Domain Containing 9/9 like (*SAMD9/SAMD9L*), RUNX family transcription factor 1 (*RUNX1*), CCAAT enhancer binding protein alpha (*CEBPA*), ETS variant transcription factor 6 (*ETV6*), DEAD-box helicase 41 (*DDX41*)*,* or Ankyrin Repeat Domain 26 *(ANKRD26*) mutations [9,10,11,12,13,14,15,16,17,18]. In general, while the incidence of primary MDS is usually higher in elderly individuals, MDS/AML related to the aforementioned disorders occur in younger patients, because of the intrinsic increased genomic instability, facilitating the acquisition of new clonal or subclonal mutations. [19,20] Specific acquired mutations may be associated with treatment resistance (*TP53* mutations) or disease progression (neuroblastoma RAS viral oncogene homologue (*NRAS*), FMS-like tyrosine kinase III (*FLT3*), Wilms’ tumor gene (*WT1*), protein tyrosine phosphatase non-receptor type 11 (*PTPN11*), isocitrate dehydrogenase (*IDH1/2*), and Nucleophosmin 1 (*NPM1*) mutations) either in primary vs. inherited contexts, and may be present before clinical changes become apparent [21,22]. Thus, monitoring patients’ genetic profile during the disease course may be helpful for clinical management, especially in selected high-risk patients.

Looking at the clonal architecture of MDS within an evolutionary perspective, different patterns may be identified, depending on whether mutations are acquired linearly until the selection of a clone with proliferative advantage (linear model), or by divergence of a different set of subclones from a common ancestral, with the coexistence of partially overlapping mutations (branching model) [8,23,24,25]. 

Many environmental forces may shape the complexity of this genomic landscape. For instance, the selective pressure created by disease-modifying therapeutic interventions may select for resistant or high-risk clones through a bottleneck effect and contribute to AML transformation. Nonetheless, immune-related signatures may influence clonal selection, and vice versa, certain mutations (e.g., EP300 and TP53) may be the cause of an altered immune environment, impacting on disease progression and treatment resistance [26].

The deep understanding of the tremendous genetic complexity is opening many attractive treatment windows for patients with MDS. Here we aim to comprehensively discuss the genetic landscape of specific molecular aspects and dig into molecular targeted therapies.

## 2. Therapeutic Strategies

### 2.1. Hypomethylating Agents

DNA methylation is one of the most affected pathways in MDS patients. DNA methyltransferase enzymes (DNMT1, DNMT3A, and DNMT3B) are responsible for the conversion of cytosine to 5-methyl-cytosine (5 mC) by adding a methyl group at the C5 position of cytosine [27]. Conversely ten-eleven translocation (TET) enzymes, TET1, TET2, and TET3 are necessary for demethylation, catalyzing the hydroxylation of 5 methylcytosine with the production of 5-hydroxy-methyl-cytosine (5 hmC) [28,29]. Mutations affecting genes encoding for those enzymes and, particularly *DNMT3A* and *TET2*, are highly frequent in MDS and AML patients, denoting their key role in the pathogenesis of myeloid neoplasms [30,31]. Moreover, an altered epigenome due to aberrant methylation is present regardless the presence of somatic mutations in epigenetic regulators, suggesting convergent pathophysiologic mechanisms and the importance of this pathway in hematopoietic development [31,32].

Hypomethylating agents (HMA) 5-azacytidine (AZA) and 5-aza-2′-deoxycytidine (decitabine, DEC), are considered the standard of care for patients with MDS. Those molecules, together with the new generation HMA 2′-deoxy-5-azacytidylyl-(3′-5′)-2′-deoxyguanosine (guadecitabine, GUA), act as DNMT inhibitors and re-start DNA demethylation by restoring the transcription of previously silenced genes [33].

AZA is a chemical analogue of the nucleoside cytosine exerting its antineoplastic activity through the direct inhibition of DNA methyltransferase. In particular, at low doses AZA generates hypomethylation while at high doses is cytotoxic because of its incorporation into DNA and RNA. This latter phenomenon leads to the disassembly of polyribosomes and inhibition of protein synthesis, causing apoptosis.

**Figure 1:**
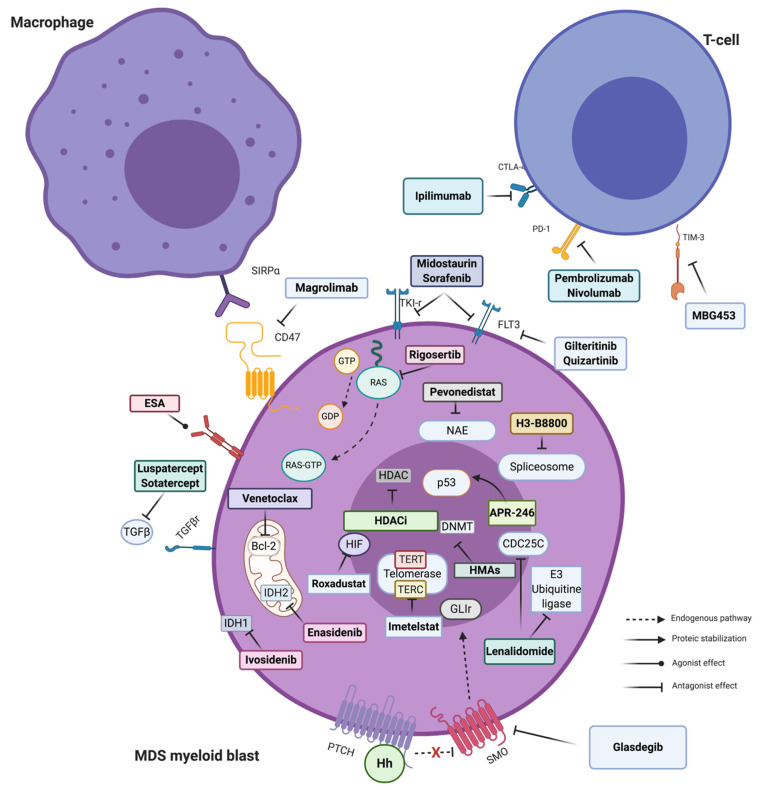
Principal targeted therapies available in MDS. Stylized pathways are also indicated. The arrow and connector style indicate the type of molecular effect. Majority of targeted drugs have an antagonist effect and work inhibiting a more or less specific enzymatic/proteic function. Exceptions are represented by erythropoietin stimulating agent (ESA) (agonists of Erythropoietin (EPO) receptor, not discussed here in detail) and APR-246, the new p53-mutated targeted agent, able to refold the aberrant protein restoring its transcriptional activity. For description of each mechanism of action see text. Figure created with BioRender. Abbreviations: SIRPα: Signal regulatory protein alpha; ESA: Erythropoietin stimulating agent; TGFβ: Transforming growth factor beta; IDH: Isocitrate dehydrogenase; HDAC(i): Histone deacetylases (inhibitor); Hh: Hedgehog polypeptides; PTCH: Protein patched homolog; SMO: Smoothened; HMA: Hypomethylating agents; DNMT: DNA methyl transferase; NAE: Neural Precursor Cell Expressed, Developmentally Down-Regulated 8 (NEDD8)-activating enzyme; GTP: guanosine triphosphate; GDP: Guanosine 5′-diphosphate; FLT3: FMS-like tyrosine kinase 3; PD-1: Programmed cell death 1; CTLA4: cytotoxic T-lymphocyte-associated protein 4; HIF: Fypoxia inducible factor; TERT: Telomerase reverse transcriptase; and TERC: Telomerase RNA component.

DEC is considered a prodrug analogous to cytosine, that is transported into cells and subsequently phosphorylated to generate the active molecule 5-aza-2′-deoxycytidine-triphosphate [33,34]. This molecule is then incorporated into DNA by DNA polymerase during DNA replication, and inhibits DNMT1 through the formation of a covalent bond [35,36]. At low concentrations, the drug depletes DNMTs with the result of a global DNA hypomethylation while at high concentrations, DEC generates double-strand breaks and cell death (Figure 1) [32,33,34].

While in the U.S. AZA and DEC are approved for all MDS patients, in Europe HMAs are approved only for high-risk categories and AML. Although both have been shown to be able to delay progression to AML, only AZA has demonstrated a significant improvement in overall survival (OS) in randomized trials compared to best supportive care [37,38,39,40]. However, subsequent data from both prospective and retrospective studies did not show the same benefits, probably because of less rigorous patient selection and adherence to treatment schedules and duration [41,42]. Overall, less than 20% of patients achieve a complete response (CR) that in most cases is transient, with a variable duration (6 to 24 months) [41,42]. Importantly HMAs may show their clinical effectiveness after a minimum of six cycles of treatment at standard doses (AZA: 75 mg/m^2^ per day for 7 days at 4-week intervals, or DEC: 20 mg/m^2^ per day for 5 days at 4-week intervals) while their early discontinuation can lead to rapid loss of response also in patients who previously achieved a complete remission [43,44,45]. Although, for eligible patients, allogeneic hematopoietic stem cell transplantation (allo-HSCT) is ideally recognized as the only potentially curative strategy, outcomes of this procedure remain dismal especially in higher risk categories after HMA failure with high relapse and treatment-related mortality rates, thus limiting the number of patients who can potentially benefit from this therapy [46,47]. In this context, given the paucity of effective salvage therapies, HMA failure remains an unmet medical need especially in patients with high-risk MDS.

**Figure 2:**
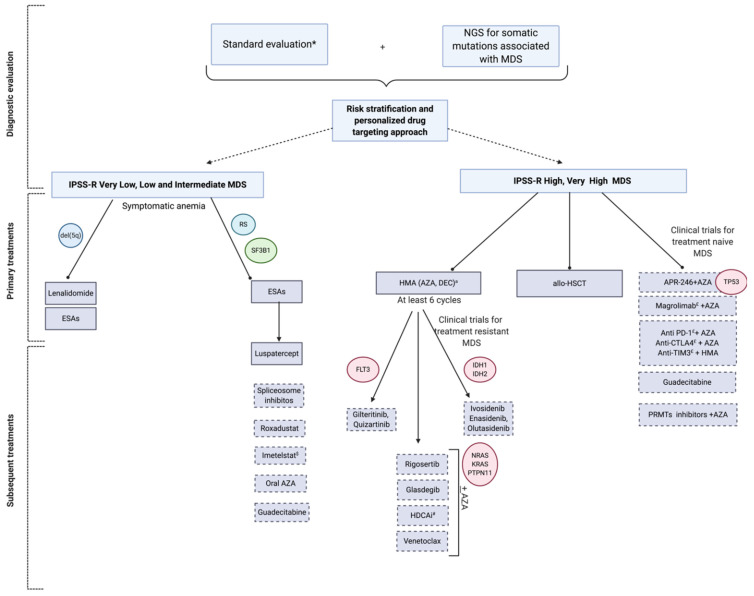
Precision management of MDS in the era of genomics and new tailored treatments. Diagnostic and therapeutic algorithm of patients with MDS according to individualized approaches taking into account the Revised International Prognostic Scoring System (IPSS-R) and genomic data in the choice of targeted treatments. * Standard evaluation includes: Complete blood count (CBC), examination of peripheral blood smear, basic chemistry panel, serological tests for CMV, EBV, HIV, viral hepatitis, PB19, haptoglobin, serum erythropoietin, folate, serum ferritin, iron, TIBC, TSH, LDH, PNH clone, bone marrow cytomorphology, cytogenetics, flow cytometry. ^a^ AZA is currently approved in U.S. for all MDS and in Europe for high risk MDS; DEC is approved in U.S. for high-risk MDS/AML and in Europe for AML. ^$^ Hypothetical benefit of telomere length assessment ^#^ Hypothetical benefit for gene testing and histone acetyltransferases. Abbreviations: MDS: Myelodysplastic syndromes, RS: Ring sideroblasts, TF: Transfusion, HDAC(i): Histone deacetylases (inhibitor); AZA: Azacitidine; DEC: Decitabine; PRMTs: Protein arginine methyltransferases; and allo-HSCT: Allogeneic stem cell transplantation; CMV: cytomegalovirus; Epstein-Barr virus, EBV; Human immunodeficiency virus, HIV; parvovirus, B19, PB19; Total iron binding capacity, TIBC; thyroid stimulating hormone, TSH; Lactate Dehydrogenase, LDH; Paroxysmal Nocturnal Hemoglobinuria, PNH; Dotted box indicate investigational drugs.

See Figure 2 for a proposed management in the era of genomics and new targeted treatments. When possible, patients should be enrolled in prospective clinical trials. With this in mind we will address when opportune at the end of the following sections the hypothetical subcategories of patients who could benefit from the off-label use of each agent, if not possible to include the patient in an investigational study.

### 2.2. New Generation HMA

Guadecitabine (SGI-110) is a new generation HMA, rationally designed as DEC analogue, resistant to deamination by cytidine deaminase, with a longer half-life compared to its previous cognates. Besides the direct effect on cancer-associated methylation patterns, several in vitro and in vivo studies have shown its potential in sensitizing tumor cells to other anticancer treatments, including immunomodulatory agents [48,49,50].

Despite promising preclinical results and encouraging data from phase I/II trials [51,52] showing overall response rates (ORR) of 51% (22% CR) and 43% (4% CR) in the frontline and HMA-refractory setting, according to the preliminary results of two phase III studies (NCT02907359 and NCT02920008, respectively), it is premature to admit an impact of guadecitabine in improving significantly the survival of MDS/chronic myelomonocytic leukemia (CMML) and AML patients [53].

Several oral formulations of HMAs are in study in MDS and AML settings for both minimizing patient discomfort and enabling a more effective pharmacodynamics. CC-486 (oral AZA) has shown acceptable toxicity and effectiveness in patients with MDS and in general myeloid malignancies, with response rates of up to 46% when given on a 21 out of 28-day schedule [54,55]. CC-486 has recently been approved by the Food and Drug Administration (FDA) under the name of Onureg^®^ as maintenance therapy in AML patients in first remission after intensive induction chemotherapy [56]. This molecule is currently in evaluation in ongoing trials as post-allo-HSCT maintenance (NCT04173533) or for patients with low-risk MDS (NCT01566695).

ASTX727, also recently FDA approved (July 2020, under the name INQOVI^®^) in patients with 1- and 2-intermediate and high-risk MDS and CMML, is a combination of oral DEC and cedazuridine, a cytidine deaminase inhibitor that prevents the DEC deactivation. Pharmacokinetics equivalence to intravenous (IV) formulations of DEC, and its clinically efficacy were presented in two recent American Society of Hematology (ASH) meetings, as results of the phase III open label ASCERTAIN trial, NCT03306264, that is studying ASTX727 for the frontline treatment of patients with high risk-MDS, CMML and AML. ORR has been reported as high as 61% (CR 21%) with a median time to CR of 4.3 months and median duration of response of 7.5 months [57,58].

New generation HMA (investigational): Rescue treatment for intermediate and high-risk patients after failure of another HMA, or in selected low-risk patients after erythropoietin stimulating agents (ESAs) failure.

Since the activity of HMAs as single agents is modest, combinatory therapies with several targeted or immunomodulatory molecules have been tested and are now in clinical development.

### 2.3. Targeted Treatments

#### 2.3.1. Venetoclax

The B-cell leukemia/lymphoma-2 (BCL-2) inhibitor, venetoclax is to date one of the most potent anti-apoptotic inhibitors, representing a novel promising agent. This small molecule blocks the binding of BH3 proteins (mostly Bim) to Bcl-2, allowing the activation of BCL2 Antagonist/Killer/BCL2 associated X (Bak/Bax) on the surface of mitochondria leading to cell death of cancer cells because of the release of cytochrome C through a mitochondrial outer membrane permeabilization (MOMP) process. The combinations of venetoclax with HMAs or low-dose cytarabine has been approved for the treatment of older or intensive chemotherapy-ineligible AML patients [59] and results from a recent published phase III randomized trial showed the superiority of AZA + Venetoclax compared to AZA + placebo in previously untreated AML patients (median survival 14.7 vs. 9.6 months and composite complete remission rates of 37% vs. 18%, respectively) [60]. A number of trials are currently evaluating the use of venetoclax, alone or in combinations with HMAs, also in MDS setting. Preliminary data from a phase I study showed a promising clinical efficacy compared to historical controls in relapsed/refractory MDS [61].

Venetoclax (investigational): Rescue treatment after HMA failure in high-risk patients, in combination with AZA. Hypothetical targeted population: High-risk treatment naïve patients, unfit for intensive chemotherapy or allogeneic HSCT.

#### 2.3.2. IDH INHIBITORS

IDH1 and 2 mutations are found in 5–10% of patients with MDS and in general are associated with worse prognosis and leukemia progression [62,63]. IDH1 and IDH2 inhibitors (ivosidenib and enasidenib) have recently shown an acceptable ORR of 42–67% in phase I and II trials [64,65] with response rates over 50% in patients with HMA failure [64]. Olutasidenib (FT-2102) is also an IDH1 inhibitor in clinical trials. Results from phase I/II trials of olutasidenib alone or in combination with AZA or cytarabine showed a ORR of 33% and 73%, respectively, as single agent or in the combinatory schema [66].

IDH inhibitors (investigational): High risk patients harboring neomorfic IDH1 or IDH2 mutations, in association with AZA, after HMA failure. Hypothetical targeted population: All risk treatment naïve IDH1/2 mutated patients, unfit for intensive chemotherapy or allogeneic HSCT.

#### 2.3.3. FLT3 Inhibitors

Pathogenic mutations in *FLT3* gene (particularly *FLT3* internal tandem duplication—(ITD)- and tyrosine kinase domain—TKD-mutations) are not a frequent event in MDS, accounting for 0.5–2% of patients at diagnosis, but when present, they are associated with high transformation rate and poor survival outcomes in the majority of cases [67,68,69,70].

FLT3 inhibitors represent another important potential group of targeted compounds in MDS patients harboring *FLT3* mutations. However, data concerning their safety and effectiveness come essentially from studies recruiting AML patients.

Midostaurin is a broad-spectrum tyrosine kinase (TK) inhibitor acting on both wild type and mutated FLT3 kynases. Alone, this compound did not show significant advantages in patients with relapsing refractory AML or high-risk MDS [71,72]. However its association with intensive chemotherapy in AML FLT3 mutated patients has been approved based on the results of a phase III randomized trial, demonstrating a significant improvement of survival in patients receiving standard chemotherapy plus midostaurin compared to chemotherapy and placebo [73]. A phase I/II trial studying its combination with AZA in AML and high-risk MDS patients ineligible to intensive chemotherapy showed more modest results, with an ORR of 26% and a median time of survival of 20 weeks [74].

Sorafenib is another multitarget FLT3 inhibitor, FDA-approved for the treatment of non-hematological malignancies such as hepatocellular, renal cell, and differentiated thyroid cancers [75] and whose efficacy has been very recently shown in prevention of disease relapse after allo-HSCT in patients with *FLT3*-ITD AML [76]. However first I/II trials using sorafenib in combination with low doses of cytarabine in high risk MDS gave disappointed results with around 10% of responses [77] whereas studies evaluating its efficacy in combination with AZA were limited to AML patients [78].

Gilteritinib is a potent second generation inhibitor of both FLT3 and AXL, an oncogenic tyrosine kinase frequently overexpressed in AML and that facilitates FLT3 activation, a known mechanism of FLT3 tyrosine kinase inhibitor (TKI) resistance [79,80]. It has been approved by FDA as single agent in relapsed/refractory AML with *FLT3* mutations [81]. However, studies evaluating its efficacy in MDS setting are still ongoing (NCT04027309, NCT04140487).

Quizartinib is another second-generation FLT3 inhibitor that has shown activity in monotherapy in the relapse and refractory AML patients demonstrating composite CR rates >40% [82,83,84]. However also in this case its efficacy is under investigation in MDS patients (NCT03661307, NCT04493138, NCT01892371).

Quizartinib and Gilteritinib (investigational): FLT3 mutated patients non-responding to HMA. Hypothetical targeted population: All high-risk FLT3 mutated treatment naïve patients, unfit for intensive chemotherapy or allogeneic HSCT.

#### 2.3.4. Splicing Inhibitors

Mutations in genes encoding for the different spliceosome components (*SF3B1*, serine/arginine-rich splicing factor 2 (*SRSF2*), U2 small nuclear RNA auxiliary factor1 (*U2AF1*), and zinc finger CCCH-type, RNA binding motif and serine/arginine rich 2 (*ZRSR2*)) are recurrent events in MDS and pathophysiologically can lead to an aberrant alternative splicing through activation of incorrect splice sites or intron retention of downstream genes, inducing functional haploinsufficient expression in some instances, similar to chromosomal deletions, epigenetic silencing, or inactivating mutations [85,86,87]. Giving their role in disease pathogenesis and the fact that they often occur in heterozygous status and in a mutually exclusive configuration, inhibition of the aberrant spliceosome machinery represents an attractive targeted approach as shown in in preclinical models [88]. Specifically, H3B-8800, a selective and orally bioavailable modulator of normal and mutant SF3b complex, has shown dose-dependent modulation of splicing in pre-clinical xenograft models [89] and is currently in study in an open-label, multicenter phase I trial for patients with previously treated MDS, AML, and CMML (NCT02841540). The discovery of H3B-8800 follow a path of research efforts on natural products. Bacterially-derivatived products have been shown to bind the SF3B complex to disrupt early stages of spliceosome cascade. These compounds have different stabilities and include low stability-agents (FR901463, FR901464, FR901465, herboxidienes, and pladienolides), and high stability-agents (E7107, a pladienolide B-analog, spliceostatin A, and sudemycins). While several compounds have only been shown to biologically alter splicing in vitro, a few compounds have been tested in both in vitro and in vivo [90,91,92]. For instance, E7107 seems to induce severe splicing inhibition in several cellular and animal models although its effects in human have not be proven. More recently cells carrying splicing factor mutations have been found sensitive to treatment with sulfonamides. Leukemia hematopoietic stem cells (HSCs) showed sensitivity to treatment with aryl sulfonamides (e.g., indisulam). In fact, drug sensitivity correlated with increased DDB1 and CUL4 associated factor 15 (DCAF15) expression levels. Indisulam and other sulfonamides seem to induce the degradation of RNA binding motif protein 39 (RBM39), leading to abnormal mRNA splicing changes (intron retention, exon skipping) [93]. The antiproliferative effect of a novel chloroindolyl sulfonamide (E7070) in combination with idarubicin and cytarabine is being investigated in relapsed AML and high-risk MDS (NCT01692197). Moreover, protein arginine methyltransferases (PRMTs) inhibitors (MS023, GSK591) influenced the growth of *SRSF2* mutant cells. The clinical activity of the PRMT5, GSK3326595, is being investigated in combination with AZA in newly-diagnosed MDS and AML (NCT03614728).

H3B-8800 and PRMTs inhibitors (investigational): Patients of all IPSS-R risks after HMA failure (phase I). Giving the early investigational phase, their use outside of a clinical trial is not recommended.

#### 2.3.5. Histone Modifiers

Heterozygous somatic mutations in genes encoding for epigenetic regulators have been found in all subgroups of myeloid disorders, and aberrant histone modifications, such as acetylation, play a key role in myeloid malignancy pathophysiology, underpinning the rationale for the use of epigenetic modifiers in AML and MDS [4,94,95]. Histone deacetylase inhibitors (HDCAi) are an interesting class of agents used also in several hematological disorders. Designed to target histone deacetylases (HDAC), these drugs can modify the expression pattern of numerous genes, including oncogenes and tumor suppressors, inducing apoptosis, differentiation, and cell cycle arrest in cancer cells, although the exact mechanism of action remains still unclear [96,97].

Although phase I and II studies evaluating those agents in monotherapy for AML and MDS patients showed negative results and absence of acceptable response rates [98,99,100,101] a number of trials have investigated and are currently studying of the combination of HDACi with several agents in myeloid neoplasms. However so far, phase II studies of various HDACi at various doses in combination with azacitidine or decitabine for MDS, CMML, and AML patients have not shown any improved clinical outcomes [102,103,104,105,106].

HDACi (investigational): High risk patients after HMA failure, in association with AZA. Hypothetical targeted population: Selected patients with aberrant acetylase mechanisms (e.g., abnormal expression or mutations of genes encoding for acetyltransferases).

#### 2.3.6. Drugs Targeting p53 Pathway

*TP53* mutations are associated with extremely poor survival outcomes and high progression rate in patients with MDS and AML. However, a breakthrough in the treatment of this dismal category derives from ongoing studies evaluating APR-246, an agent able to refold the mutant p53 protein, restoring its transcriptional function. This molecule already showed auspicious preclinical results in combination with AZA in vitro and in vivo models of AML/MDS. [107] Two parallel French and US-based phase Ib/II trials are evaluating this combination in HMA-naïve, *TP53*-mutated patients with high-risk MDS, CMML, or AML, and preliminary results have been highly promising with ORR between 54 and 88%, in a very high-risk elderly population, generally characterized by complex karyotype and unresponsiveness to standard treatments [108,109]. A registration phase III clinical trial comparing AZA + APR-246 with AZA in monotherapy (NCT03745716), as well as a phase II study evaluating the same combination as maintenance therapy after allo-HSCT (NCT03931291) in *TP53* mutated AML/MDS patients are ongoing.

APR-246 (investigational): High-risk treatment naïve patients harboring TP53 mutations. Hypothetical targeted population: All risk categories of patients with TP53 mutations, including after HMA failure.

### 2.4. Immunomodulatory Treatments

#### 2.4.1. Lenalidomide

Lenalidomide is a thalidomide analog with a complex mechanism of action that has demonstrated its clinical activity in low to intermediate-risk MDS patients with 5q-. This molecule in fact not only exerts a direct anti-proliferative effect on leukemia blasts, by inhibition of the cell cycle arrest and apoptosis, but is also able of a modulatory effect on immune cells of the bone marrow microenvironment in patients with MDS. Recent studies have also shown that lenalidomide acts through a karyotype-dependent manner modulating the expression of genes already haploinsufficient [110,111,112,113]. A number of phase II and III trials have evaluated this agent with various treatment schedules and associations in MDS with or without 5q-. While very satisfying results were seen when given in monotherapy in low risk/5q- setting, with response rates/hematological improvements in >60–80% of patients [114,115,116,117], results in intermediate/high risk setting were dismal with ORR of 10–30% [118,119,120]. Thus notwithstanding, a therapeutic benefit has been shown, in combinations with AZA, in intermediate and high risk MDS patients, with or without 5q-, with a significant increase in ORR and CR compared with AZA alone (ORR/CR 72/44% vs. 49/17%) in a phase II trial [121].

Lenalidomide: Approved for low-risk patients with 5q- MDS.

#### 2.4.2. Immune Checkpoint Inhibitors

Aberrant immune function and dysregulation of immune checkpoint (IC) molecules represent a known pathophysiological aspect of MDS, providing the rationale for studying the role of IC inhibitors (ICI) in those patients, especially after HMA failure [122,123,124].

The programmed cell death 1 (PD-1) inhibitor pembrolizumab and the cytotoxic T-lymphocyte-associated protein 4 (CTLA4) inhibitor, ipilimumab, have been both studied in phase Ib/II trials in patients with intermediate/high-risk MDS who failed HMA showing limited efficacy in monotherapy (interim analysis for pembrolizumab, NCT03094637) [125,126]. A phase II study, evaluating the combination of nivolumab (anti-PD-1) or ipilimumab with or without AZA, and is showing promising preliminary results, with an increased response rate in patients treated with the combinatory regimens (ORR 75, 71, 35, and 13%, in patients receiving AZA + nivolumab, AZA + ipilimumab, ipilimumab alone, or nivolumab alone, respectively, trial: NCT02530463) [127]. In contrast, results from an ongoing large phase II randomized trial (NCT02775903), that is evaluating untreated patients with high-risk MDS and AML ineligible for intensive chemotherapy to AZA + durvalumab (another programmed death-ligand 1 (PD-L1) inhibitor) or AZA alone have been unsatisfactory without clinically meaningful difference in efficacy between the two treatment arms [128].

T-cell immunoglobulin mucin (TIM-3) is another emerging target in immunotherapy, present on the surface of effector T-cells, whose hyperexpression and hyperactivation has been shown able to induce T-cell exhaustion in several cancers including myeloid malignancies [129,130,131]. MBG453 is a TIM-3 antibody that is currently in study in several disease settings. Association with DEC in a phase I study of HMA-naïve, high-risk MDS patients showed CR of 50% (preliminary results NCT03066648) [132]. Ongoing studies are evaluating other combinatory schema with AZA (NCT03946670, NCT03940352) in higher risk MDS.

CD47, a surface protein normally intervening in the recognition of “self” toward the innate immune surveillance, has been shown upregulated on the surface of cancer cells and leukemic blasts as mechanism of immune-evasion from anti-tumor macrophagic responses [133,134,135]. Magrolimab is an investigational anti-CD47 monoclonal antibody which recently received a breakthrough therapy designation from FDA, based on the positive results of a phase Ib study evaluating its association with AZA and showing a ORR of 92% (CR 50%) in previously untreated intermediate and high-risk MDS patients [136]. A double blind phase III multicenter randomized study (AZA + Magrolimab vs. AZA + placebo) has recently been registered for treatment-naive high-risk MDS patients (ENHANCE trial, NCT04313881).

Immune checkpoint inhibitors (investigational): High-risk MDS treatment naïve patients. Hypothetical targeted population: Subgroup of patients whose immune-transcriptomic profile is characterized by high expression of immune checkpoint molecules.

#### 2.4.3. Anti-TGF β

Transforming growth factor-beta (TGF β) signaling is a complex pathway whose role has been demonstrated in carcinogenesis and cancer progression as well as in the pathogenesis of a variety of hematological disorders [137,138]. Two TGF β pathway inhibitors, luspatercept [139] and sotatercept [140], have proved their efficacy in improving anemia in lower-risk MDS patients, with hematological improvement rates around 60% in two phase II studies. Of note in the sotatercept study half of the patients had a previous HMA failure [140] suggesting that those compounds may rescue severely anemic HMA-pretreated lower-risk MDS patients who otherwise would have very few therapeutic opportunities. Results of a randomized phase III trial evaluating luspatercept vs. placebo in lower risk MDS with ring sideroblasts (RS) have been published last year and showed a significant rate of transfusion independence in 38% vs. 13% of patients (*p* < 0.001) with limited adverse effects (MEDALIST trial) [141]. Based on those results luspatercept has been recently FDA approved in this setting of patients [142]. A phase III trial evaluating the efficacy of luspatercept vs. epoietin alpha in lower risk MDS, as well as a phase Ib/II study combining this anti-TGF β and lenalidomide in low-risk non-del(5q) diseases, are ongoing (NCT03682536, NCT04539236).

Luspatercept (approved): Low-risk MDS-RS with >15%of RS or with >5% of RS and SF3B1 mutation, after ESAs failure.

### 2.5. Other Targeted Therapies

Rigosertib is an oral multikinase inhibitor that acts as a RAS-mimetic binding to the proto-oncogene, serine/threonine kinase (RAF) and phosphoinositide-3-kinase (PI3K) family proteins and disrupting their ability to bind to RAS [143,144]. What is of interest for this compound is that, while it has a potent antimitotic and antineoplastic effect, it seems relatively non-toxic for normal cells. Despite encouraging results of phase I trials in MDS patients [144,145,146] with ORR of up to 53%, a randomized phase III trial of a large cohort of high risk patients refractory to HMA failed to show the superiority of rigosertib compared to best supportive therapies [147]. A synergistic effect has been seen in association with AZA, based on the results of a phase II study showing responses of 60–90% in HMA-refractory and naïve settings respectively. This combination is currently under investigation in a randomized phase III trial vs. AZA alone in treatment-naïve high-risk MDS patients [148].

Rigosertib (investigational): High risk treatment naïve patients in combination with AZA. Hypothetical targeted population: Subgroup of patients with an oncogenic or hyperactivated RAS pathway (e.g., in case of PTPN11, NRAS, KRAS, and RIT1-α gain of function mutations).

Glasdegib is a potent and selective oral inhibitor of the Hedgehog signaling pathway that showed its clinical activity in AML and MDS, particularly in combination with chemotherapy. The results of phase II studies evaluating safety and efficacy of glasdegib combined with chemotherapy in treatment naive patients with AML or high-risk MDS have been so promising that the FDA approved this agent in combination with low doses of cytarabine for the frontline treatment of elderly AML patients not eligible to intensive chemotherapy [149,150]. Results of glasdegib in monotherapy in refractory MDS patients were disappointing, with less than 10% of ORR [151]. However, a trial evaluating its efficacy in combinatory schemes with AZA is ongoing (NCT02367456).

Glasdegib (investigational): High risk treatment naïve patients in combination with AZA.

Pevonedistat (MLN4924) is a novel inhibitor of NEDD8-activating enzyme (NAE) able to induce aberrant proteosomal degradation of intracellular proteins leading to their cytotoxic accumulation and apoptosis [152]. It is currently under investigation in a phase I/II trials in combination with AZA in MDS patients refractory to HMA therapy. Interim results were encouraging, showing an ORR of about 40–70% with significant benefits especially for the high-risk MDS category [153,154]. Given these promising data the randomized phase III PANTHER trial is already ongoing and testing pevonedistat + AZA vs. AZA monotherapy for upfront treatment of high-risk-MDS, CMML, and AML patients (NCT03268954).

Pevonedistat (investigational): High-risk patients in combination with AZA after HMA failure.

Roxadustat (FG-4592) is an orally administered hypoxia inducible factor prolyl hydroxylase inhibitor (HIF-PHIs), currently FDA approved for anemia in chronic kidney disease (CKD), based on the results of a recently published phase III study [155]. This drug improves erythropoiesis by at least two mechanisms: (i) increasing endogenous erythropoietin levels and (ii) regulating iron metabolism, through downregulation of hepcidin [156]. A phase III randomized double-blind placebo-controlled trial, is analyzing the efficacy and safety of roxadustat to treat anemia in patients with lower-risk MDS and low RBC transfusion burden (NCT03263091).

Roxadustat (investigational): Very low, low and intermediate-risk patients. Hypothetical targeted population: MDS patients of all risk with multifactorial anemia due for instance to concomitant CKD.

Imetelstat is a novel, first-in-class telomerase inhibitor, targeting selectively cells with short telomere length, binding selectively the telomerase RNA component (TERC) subunit, resulting in direct, competitive inhibition of telomerase enzymatic activity [157]. Data from a phase II/III study show a reduction in disease burden in low risk patients refractory or relapsed after ESA usage [158] The phase III part of the study is still ongoing (NCT02598661) and meanwhile Imetelstat has been granted a Fast Track designation FDA for non-del(5q) lower risk MDS refractory or resistant to ESAs.

Imetelstat (investigational): Non-del(5q) low-risk patients refractory to ESAs. Hypothetical targeted population: Patients of all risks with shorter telomere length.

SY-1425 is an oral first-in-class selective retinoic acid receptor alpha (RARα) agonist investigated as maintenance therapy or in refractory acute promyelocytic leukemia [159]. Promising results have been presented at the 62nd American Society of Hematology meeting in naïve unfit AML patients (ORR 67% and 38% in patients RARα+ and RARα-) [160]. Based on the reported data, a Phase III trial in newly diagnosed high risk MDS has been announced.

A summary of all phase III studies discussed here for both approved and investigational targeted treatments is reported in Table 2.

## 3. Conclusions

While for more than a decade the therapeutic scenario of MDS syndromes has been dominated by very few available regimens associated with dismal results especially for patients with higher risk disease and who failed HMAs, in the last 2 years we assisted to the flourishing of an incredible variety of new targeted agents and investigational approaches that possibly will reach the clinical setting in short time. If current available therapies still include HMAs, lenalidomide, growth factor support, chemotherapy, and allo-HSCT (this last as unique curative solution), we have now the possibility to choose among new investigational treatments rationally designed to exert targeted actions and increase the response rates in categories of patients that so far remained precision drug-orphans. Only in 2020 luspatercept and the oral HMA ASTX727 were approved in USA while Pevonedistat and Magrolimab were just granted the designation of breakthrough therapies, which hopefully can accelerate their authorization process.

In this context, where a wide range of molecules is now becoming available, predictive and prognostic genetic markers may potentially allow for a more individualized approach either in treatment naïve or after HMA failure MDS patients, by improving risk stratification and helping in the identification of more appropriate treatment choices.

## Figures and Tables

**Table 1:** Genes recurrently mutated in primary or secondary myelodysplastic syndromes (MDS).

Pathway/Functions	Gene Name
DNA methylation	TET2, DNMT3A, IDH1, IDH2
Histone modification	ASXL1, KMT2, EZH2, SUZ12, JARID2, KDM6A, PHF6, EED, EP300
RNA splicing	SF3B1, SRSF2, U2AF1, U2AF2, ZRSR2, SF1, PRPF8, LUC7L2, DDX41
Cohesin complex	STAG2, RAD21, SMC3, SMC1A
Transcription factors	RUNX1, ETV6, GATA2, CUX1 IRF1, CEBPA, BCOR
Signal transduction	PTPN11, NF1, NRAS, KRAS, JAK2, MPL, KIT, FLT3, JAK2, CALR, CSF3R, CBL
p53 pathway	TP53, PPM1D
DNA repair	ATM, FANCA-L, BRCA2, RAD51
Others	NPM1, SETBP1, WT1

Abbreviations: TET2: Ten-eleven translocation-2; DNMT: DNA methyl transferase; IDH: Isocitrate dehydrogenase; ASXL1: ASXL transcriptional regulator 1; KMT2: Histone–lysine N-methyltransferase; EZH2: Enhancer of zeste homolog 2; SUZ12: SUZ12 polycomb repressive complex 2 subunit; JARID2: Jumonji, AT rich interactive domain 2; KDM6A: Lysine demethylase 6A; PHF6: plant homeodomain (PHD) finger protein 6; EED: Embryonic ectoderm development protein; EP300: E1A binding protein P300; SF3B1: Splicing factor 3b subunit 1; SRSF2: Serine/arginine-rich splicing factor 2; U2F1/2: U2 small nuclear RNA auxiliary factor1/2; ZRSR2: Zinc finger CCCH-type, RNA binding motif and serine/arginine rich 2; SF1: Splicing factor 1; PRPF8: Pre-MRNA processing factor 8; LUC7L2: LUC7 like 2, pre-MRNA splicing factor; STAG2: Stromal antigen 2; SMC3: Structural maintenance of chromosomes 3; RUNX1: Runt-Related Transcription Factor (RUNX) family transcription factor 1; ETV6: ETS variant transcription factor 6; GATA2: GATA binding protein 2; CEBPA: CCAAT enhancer binding protein alpha; IRF1: Interferon regulatory factor 1; BCOR: BCL6 corepressor; JAK2: Janus kinase 2; MPL: MPL proto-oncogene; CALR: Calreticuline; CSF3R: Colony stimulating factor 3 receptor; PTPN11: Protein tyrosine phosphatase non-receptor type 11; NF1: Neurofibromin 1; NRAS: neuroblastoma RAS viral oncogene homologue; CBL: Cbl proto-oncogene; PPM1D: Protein phosphatase, Mg2+/Mn2+ dependent 1D; ATM: Ataxia telangiectasia mutated; BRCA2: Breast cancer type 2 susceptibility protein; NPM1: Nucleophosmin 1; SETBP1: SET binding protein 1; WT1: Wilms’ tumor gene; DDX41: DEAD-box helicase 41; FLT3: FMS-like tyrosine kinase 3; PD-1: Programmed cell death 1; and CTLA4: Cytotoxic T-lymphocyte-associated protein 4.

**Table 2:** Selected phase III studies for investigational agents in MDS patients.

Agent	Mechanism of Action	NCT	Patient Population	Study Design	Start Date	Status and Outcomes	Reference
Guadecitabine	New generation DNMT inhibitor, resistant to cytidine deaminase	NCT02907359 (ASTRAL-3 trial)	MDS, CMML after treatment failure	Multicenter, randomized, open label; Guadecitabine vs. treatment of choice (low-dose cytarabine, BSC, intensive chemotherapy)	September 2016	Ongoing study	
ASTX727	New generation DNMT inhibitor, combination of DEC and cedazuridine	NCT03306264 (ASCERTAIN trial)	Treatment naive and R/R HR-MDS, CMML, or AML	Multicenter, randomized, open label; ASTX727 vs. IV DEC	October 2017	Ongoing study	
CC-486	New generation DNMT inhibitor, oral AZA	NCT01566695	LR-MDS with transfusion-dependent Anemia and Thrombocytopenia	Multicenter, randomized, double-blind CC-486 + BSC vs. placebo + BSC	April 2012	Ongoing study	
		NCT04173533	AML or HR-MDS in CR following allogeneic HSCT	Multicenter, randomized, double-blind CC-486 vs. placebo for maintenance after allo-HSCT	November 2019	Ongoing study	
Venetoclax	Bcl-2 inhibitor	NCT04628026	Newly diagnosed AML and MDS-EB2	Multicenter, randomized, double-blind, Venetoclax + chemotherapy vs. placebo +chemotherapy	November 2020	Ongoing study	
		NCT04401748	Newly diagnosed HR-MDS	Multicenter, randomized, double-Blind, Venetoclax + AZA vs. Placebo + AZA	May 2020	Ongoing study	
Ivosidenib or Enasidenib	IDH1/2 inhibitors	NCT03839771	Newly diagnosed AML and MDS-EB2 with IDH1/2 mutations	Multicenter, double-blind, randomized, Ivosidenib or Enasidenib + chemotherapy vs. Placebo + cheotherapy	February 2019	Ongoing study	
Midostaurin or Gilteritinib	FLT3 inhibitor	NCT04027309	Newly diagnosed AML and MDS-EB2 with FLT3 mutations	Multicenter, open-label, randomized Midostaurin + chemotherapy vs. Gilteritinib + chemotherapy (followed by maintenance for 1 year according to the treatment arm)	July 2019	Ongoing study	
APR-246	p53 stabilizer	NCT03745716	Newly diagnosed MDS with TP53 mutation	multicenter, randomized, APR-246 + AZA or AZA alone	November 2018	Ongoing study	
Lenalidomide	complex (immunomodulation and cell cicle arrest)	NCT01243476	LR and Int-1 IPSS MDS with 5q- and anemia without the need of transfusion	Multicenter, randomized, double-blind Lenalidomide vs. Placebo	November 2010	Ongoing study	
		NCT01029262 (MDS-005 trial)	LR and Int-1 IPSS, transfusion dependent MDS without 5q-	Multicenter, randomized, double-blind Lenalidomide vs. Placebo	December 2009	Teminated, Primary endpoint met: TI in 27% (Lenalidomide) vs. 2.5% (Placebo), *p* < 0.01	Santini et al. JCO 2016 [117]
		NCT00843882	LR and Int-1 IPSS MDS	Multicenter, randomized, Lenalidomide vs. Lenalidomide + Epoietin alpha (Procrit)	December 2009	Terminated study (results not published yet)	
MBG453	Anti-TIM3	NCT04266301 (STIMULUS-MDS2 trial)	Treatment-naïve intermediate, high or very HR IPSS-R MDS and CMML-2	Multicenter, randomized, double-blind MBG453 + AZA vs. Placebo + AZA	February 2020	Ongoing study	
Magrolimab	Anti-CD47	NCT04313881 (ENHANCE trial)	Treatment-naïve intermediate, high or very high-risk IPSS-R MDS	Multicenter, randomized, double-blind Magrolimab + AZA vs. Placebo + AZA	March 2020	Ongoing study	
Luspatercept	Anti-TGFbeta	NCT02631070	Very low, low and Intermediate-1 IPSS-R MDS in patients with transfusion dependent anemia	Multicenter, randomized, double-blind Luspatercept vs. placebo	December 2015	Terminated, Primary endpoint met; TI in 38% (Luspatercept) vs. 13% (Placebo), *p* < 0.001	Fenaux et al. NEJM 2020 [141]
		NCT03682536 (COMMAND trial)	Very low, low and Intermediate-1 IPSS-R MDS ESA Naïve patients with transfusion dependent anemia	Multicenter, randomized, open label Luspatercept vs. Epoietin alpha	September 2018	Ongoing study	
Rigosertib	Multikinase inhibitor	NCT01241500 (ONTIME trial)	MDS-EB1/2 after HMA failure	Multicenter, randomized, open label Rigosertib vs. BSC	December 2010	Terminated. (Preliminary analysis: Failure in meeting the primary endpoint. Median OS rigosertib 8.2 months vs. 5.9 months with BSC (HR 0.87, 95% CI 0.67–1.14; *p* = 0.33).	Garcia-Manero et al. Lancet Onc. 2016 [147]
		NCT02562443 (INSPIRE trial)	very HR IPSS-R MDS after HMA failure	Multicenter, randomized, open label; Rigosertib vs. BSC	September 2015	Ongoing study	
Pevonedistat	NAE inhibitor	NCT03268954 (PANTHER trial)	Newly diagnosed HR-MDS, CMML, or pauciblastic AML	Multicenter, randomized, open-label; Pevonedistat + AZA vs. AZA alone	August 2017	Ongoing study	
Imetelstat	Telomerase inhibitor	NCT02598661	Low and Intermediate-1 IPSS-R MDS ESA resistant/refractory patients	Mutlicenter phase 2/3 composed study (phase2: Open label signle arm; phase 3: Double-blind, randomized, Imetelstat vs. placebo)	November 2015	Phase 2 published/Phase 3 ongoing	Steensma et al., JCO 2021 [158]
Roxadustat (FG4592)	HIF inhibitor	NCT03263091	Primary MDS (Very Low, Low or Intermediate IPSS-R with <5% blasts)	Multicenter, randomized double-blind; Roxadustat vs. placebo	August 2017	Ongoing study	

Abbreviations: MDS: Myelodysplastic syndrome, HR: High risk; LR: Low risk; EB: Excess blast; Int: Intermediate; CMML: Chronic myelomonocytic leukemia; AML: Acute myeloid leukemia; AZA: 5-Azacytidine; DEC: Decytabine; BSC: Best supportive care; ESA: Erythropoietin stimulating agents; TI: Transfusion independence; R/R: Relapsed/refractory; NAE: NEDD8-activating enzyme; and HIF: Hypoxia inducible factor.

## References

[1] Arber D.A., Orazi A., Hasserjian R., Thiele J., Borowitz M.J., Le Beau M.M., Bloomfield C.D., Cazzola M., Vardiman J.W. (2016). The 2016 revision to the World Health Organization classification of myeloid neoplasms and acute leukemia. Blood.

[2] Kubasch A.S., Platzbecker U. (2020). Patient stratification in myelodysplastic syndromes: How a puzzle may become a map. Hematol. Am. Soc. Hematol Educ Program..

[3] Yun S., Geyer S.M., Komrokji R.S., Al Ali N.H., Song J., Hussaini M., Sweet K.L., Lancet J.E., List A.F., Padron E. (2020). Prognostic significance of serial molecular annotation in myelodysplastic syndromes (MDS) and secondary acute myeloid leukemia (sAML). Leukemia.

[4] Papaemmanuil E., Gerstung M., Malcovati L., Tauro S., Gundem G., Van Loo P., Yoon C.J., Ellis P., Wedge D.C., Pellagatti A. (2013). Clinical and biological implications of driver mutations in myelodysplastic syndromes. Blood.

[5] Haferlach T., Nagata Y., Grossmann V., Okuno Y., Bacher U., Nagae G., Schnittger S., Sanada M., Kon A., Alpermann T. (2014). Landscape of genetic lesions in 944 patients with myelodysplastic syndromes. Leukemia.

[6] Cazzola M., Della Porta M.G., Malcovati L. (2013). The genetic basis of myelodysplasia and its clinical relevance. Blood.

[7] Ogawa S. (2020). Genetic basis of myelodysplastic syndromes. Proc. Jpn. Acad Ser. B Phys. Biol. Sci..

[8] Da Silva-Coelho P., Kroeze L.I., Yoshida K., Koorenhof-Scheele T.N., Knops R., van de Locht L.T., de Graaf A.O., Massop M., Sandmann S., Dugas M. (2017). Clonal evolution in myelodysplastic syndromes. Nat. Commun..

[9] Boocock G.R.B., Morrison J.A., Popovic M., Richards N., Ellis L., Durie P.R., Rommens J.M. (2003). Mutations in SBDS are associated with Shwachman–Diamond syndrome. Nat. Genet..

[10] Niewisch M.R., Savage S.A. (2019). An update on the biology and management of dyskeratosis congenita and related telomere biology disorders. Expert Rev. Hematol..

[11] Niemeyer C.M. (2014). RAS diseases in children. Haematologica.

[12] Freedman M.H., Bonilla M.A., Fier C., Bolyard A.A., Scarlata D., Boxer L.A., Brown S., Cham B., Kannourakis G., Kinsey S.E. (2000). Myelodysplasia syndrome and acute myeloid leukemia in patients with congenital neutropenia receiving G-CSF therapy. Blood.

[13] Germeshausen M., Ballmaier M., Welte K. (2007). Incidence of CSF3R mutations in severe congenital neutropenia and relevance for leukemogenesis: Results of a long-term survey. Blood.

[14] Quentin S., Cuccuini W., Ceccaldi R., Nibourel O., Pondarre C., Pagès M.-P., Vasquez N., Dubois d’Enghien C., Larghero J., Peffault de Latour R. (2011). Myelodysplasia and leukemia of Fanconi anemia are associated with a specific pattern of genomic abnormalities that includes cryptic RUNX1/AML1 lesions. Blood.

[15] Bezzerri V., Cipolli M. (2019). Shwachman-Diamond Syndrome: Molecular Mechanisms and Current Perspectives. Mol. Diagn. Ther..

[16] Zhang M.Y., Churpek J.E., Keel S.B., Walsh T., Lee M.K., Loeb K.R., Gulsuner S., Pritchard C.C., Sanchez-Bonilla M., Delrow J.J. (2015). Germline ETV6 mutations in familial thrombocytopenia and hematologic malignancy. Nat. Genet..

[17] Poggi M., Canault M., Favier M., Turro E., Saultier P., Ghalloussi D., Baccini V., Vidal L., Mezzapesa A., Chelghoum N. (2017). Germline variants in ETV6 underlie reduced platelet formation, platelet dysfunction and increased levels of circulating CD34+ progenitors. Haematologica.

[18] Sarasin A., Quentin S., Droin N., Sahbatou M., Saada V., Auger N., Boursin Y., Dessen P., Raimbault A., Asnafi V. (2019). Familial predisposition to TP53/complex karyotype MDS and leukemia in DNA repair-deficient xeroderma pigmentosum. Blood.

[19] Babushok D.V., Bessler M., Olson T.S. (2016). Genetic predisposition to myelodysplastic syndrome and acute myeloid leukemia in children and young adults. Leuk Lymphoma.

[20] Bannon S.A., DiNardo C.D. (2016). Hereditary Predispositions to Myelodysplastic Syndrome. Int. J. Mol. Sci..

[21] Makishima H., Yoshizato T., Yoshida K., Sekeres M.A., Radivoyevitch T., Suzuki H., Przychodzen B., Nagata Y., Meggendorfer M., Sanada M. (2017). Dynamics of clonal evolution in myelodysplastic syndromes. Nat. Genet..

[22] Walter M.J., Shen D., Ding L., Shao J., Koboldt D.C., Chen K., Larson D.E., McLellan M.D., Dooling D., Abbott R. (2012). Clonal architecture of secondary acute myeloid leukemia. N. Engl. J. Med..

[23] Davis A., Gao R., Navin N. (2017). Tumor evolution: Linear, branching, neutral or punctuated?. Biochim. Et Biophys. Acta Rev. Cancer.

[24] Welch J.S., Ley T.J., Link D.C., Miller C.A., Larson D.E., Koboldt D.C., Wartman L.D., Lamprecht T.L., Liu F., Xia J. (2012). The origin and evolution of mutations in acute myeloid leukemia. Cell.

[25] Grove C.S., Vassiliou G.S. (2014). Acute myeloid leukaemia: A paradigm for the clonal evolution of cancer?. Dis. Model. Mech.

[26] Sallman D.A., McLemore A.F., Aldrich A.L., Komrokji R.S., McGraw K.L., Dhawan A., Geyer S., Hou H.-A., Eksioglu E.A., Sullivan A. (2020). TP53 mutations in myelodysplastic syndromes and secondary AML confer an immunosuppressive phenotype. Blood.

[27] Schulze I., Rohde C., Scheller-Wendorff M., Bäumer N., Krause A., Herbst F., Riemke P., Hebestreit K., Tschanter P., Lin Q. (2016). Increased DNA methylation of Dnmt3b targets impairs leukemogenesis. Blood.

[28] Tahiliani M., Koh K.P., Shen Y., Pastor W.A., Bandukwala H., Brudno Y., Agarwal S., Iyer L.M., Liu D.R., Aravind L. (2009). Conversion of 5-Methylcytosine to 5-Hydroxymethylcytosine in Mammalian DNA by MLL Partner TET1. Science.

[29] Ito S., Shen L., Dai Q., Wu S.C., Collins L.B., Swenberg J.A., He C., Zhang Y. (2011). Tet Proteins Can Convert 5-Methylcytosine to 5-Formylcytosine and 5-Carboxylcytosine. Science.

[30] Walter M.J., Ding L., Shen D., Shao J., Grillot M., McLellan M., Fulton R., Schmidt H., Kalicki-Veizer J., O’Laughlin M. (2011). Recurrent DNMT3A mutations in patients with myelodysplastic syndromes. Leukemia.

[31] Abdel-Wahab O., Mullally A., Hedvat C., Garcia-Manero G., Patel J., Wadleigh M., Malinge S., Yao J., Kilpivaara O., Bhat R. (2009). Genetic characterization of TET1, TET2, and TET3 alterations in myeloid malignancies. Blood.

[32] Seelan R.S., Mukhopadhyay P., Pisano M.M., Greene R.M. (2018). Effects of 5-Aza-2’-deoxycytidine (decitabine) on gene expression. Drug Metab Rev..

[33] Stresemann C., Lyko F. (2008). Modes of action of the DNA methyltransferase inhibitors azacytidine and decitabine. Int. J. Cancer.

[34] Daskalakis M., Blagitko-Dorfs N., Hackanson B. (2010). Decitabine. Recent Results Cancer Res..

[35] Ueda K., Masuda A., Fukuda M., Tanaka S., Hosokawa M., Iwakawa S. (2017). Monophosphorylation by deoxycytidine kinase affects apparent cellular uptake of decitabine in HCT116 colon cancer cells. Drug Metab. Pharmacokinet..

[36] Oki Y., Aoki E., Issa J.-P.J. (2007). Decitabine—Bedside to bench. Crit. Rev. Oncol. Hematol..

[37] Silverman L.R., Demakos E.P., Peterson B.L., Kornblith A.B., Holland J.C., Odchimar-Reissig R., Stone R.M., Nelson D., Powell B.L., DeCastro C.M. (2002). Randomized controlled trial of azacitidine in patients with the myelodysplastic syndrome: A study of the cancer and leukemia group B. J. Clin. Oncol.

[38] Fenaux P., Mufti G.J., Hellstrom-Lindberg E., Santini V., Finelli C., Giagounidis A., Schoch R., Gattermann N., Sanz G., List A. (2009). Efficacy of azacitidine compared with that of conventional care regimens in the treatment of higher-risk myelodysplastic syndromes: A randomised, open-label, phase III study. Lancet Oncol..

[39] Lübbert M., Suciu S., Baila L., Rüter B.H., Platzbecker U., Giagounidis A., Selleslag D., Labar B., Germing U., Salih H.R. (2011). Low-Dose Decitabine Versus Best Supportive Care in Elderly Patients With Intermediate- or High-Risk Myelodysplastic Syndrome (MDS) Ineligible for Intensive Chemotherapy: Final Results of the Randomized Phase III Study of the European Organisation for Research and Treatment of Cancer Leukemia Group and the German MDS Study Group. J. Clin. Oncol..

[40] Kantarjian H., Issa J.-P.J., Rosenfeld C.S., Bennett J.M., Albitar M., DiPersio J., Klimek V., Slack J., de Castro C., Ravandi F. (2006). Decitabine improves patient outcomes in myelodysplastic syndromes: Results of a phase III randomized study. Cancer.

[41] Bernal T., Martínez-Camblor P., Sánchez-García J., de Paz R., Luño E., Nomdedeu B., Ardanaz M.T., Pedro C., On behalf of The Spanish Group on Myelodysplastic Syndromes and PETHEMA Foundation, Spanish Society of Hematology (2015). Effectiveness of azacitidine in unselected high-risk myelodysplastic syndromes: Results from the Spanish registry. Leukemia.

[42] Dinmohamed A.G., van Norden Y., Visser O., Posthuma E.F.M., Huijgens P.C., Sonneveld P., van de Loosdrecht A.A., Jongen-Lavrencic M. (2015). Effectiveness of azacitidine for the treatment of higher-risk myelodysplastic syndromes in daily practice: Results from the Dutch population-based PHAROS MDS registry. Leukemia.

[43] Greenberg P.L., Stone R.M., Al-Kali A., Barta S.K., Bejar R., Bennett J.M., Carraway H., De Castro C.M., Deeg H.J., DeZern A.E. (2017). Myelodysplastic Syndromes, Version 2.2017, NCCN Clinical Practice Guidelines in Oncology. J. Natl. Compr. Cancer Netw..

[44] Silverman L.R., Fenaux P., Mufti G.J., Santini V., Hellström-Lindberg E., Gattermann N., Sanz G., List A.F., Gore S.D., Seymour J.F. (2011). Continued azacitidine therapy beyond time of first response improves quality of response in patients with higher-risk myelodysplastic syndromes. Cancer.

[45] Voso M.T., Breccia M., Lunghi M., Poloni A., Niscola P., Finelli C., Bari A., Musto P., Zambello R., Fianchi L. (2013). Rapid loss of response after withdrawal of treatment with azacitidine: A case series in patients with higher-risk myelodysplastic syndromes or chronic myelomonocytic leukemia. Eur. J. Haematol..

[46] Festuccia M., Baker K., Gooley T.A., Sandmaier B.M., Deeg H.J., Scott B.L. (2017). Hematopoietic Cell Transplantation in Myelodysplastic Syndromes after Treatment with Hypomethylating Agents. Biol. Blood Marrow Transpl..

[47] Damaj G., Duhamel A., Robin M., Beguin Y., Michallet M., Mohty M., Vigouroux S., Bories P., Garnier A., El Cheikh J. (2012). Impact of Azacitidine Before Allogeneic Stem-Cell Transplantation for Myelodysplastic Syndromes: A Study by the Société Française de Greffe de Moelle et de Thérapie-Cellulaire and the Groupe-Francophone des Myélodysplasies. J. Clin. Oncol..

[48] Kuang Y., El-Khoueiry A., Taverna P., Ljungman M., Neamati N. (2015). Guadecitabine (SGI-110) priming sensitizes hepatocellular carcinoma cells to oxaliplatin. Mol. Oncol..

[49] Srivastava P., Paluch B.E., Matsuzaki J., James S.R., Collamat-Lai G., Karbach J., Nemeth M.J., Taverna P., Karpf A.R., Griffiths E.A. (2014). Immunomodulatory action of SGI-110, a hypomethylating agent, in acute myeloid leukemia cells and xenografts. Leuk Res..

[50] Fang F., Munck J., Tang J., Taverna P., Wang Y., Miller D.F.B., Pilrose J., Choy G., Azab M., Pawelczak K.S. (2014). The novel, small-molecule DNA methylation inhibitor SGI-110 as an ovarian cancer chemosensitizer. Clin. Cancer Res..

[51] Garcia-Manero G., Roboz G., Walsh K., Kantarjian H., Ritchie E., Kropf P., O’Connell C., Tibes R., Lunin S., Rosenblat T. (2019). Guadecitabine (SGI-110) in patients with intermediate or high-risk myelodysplastic syndromes: Phase 2 results from a multicentre, open-label, randomised, phase 1/2 trial. Lancet Haematol..

[52] Kantarjian H.M., Roboz G.J., Kropf P.L., Yee K.W.L., O’Connell C.L., Tibes R., Walsh K.J., Podoltsev N.A., Griffiths E.A., Jabbour E. (2017). Guadecitabine (SGI-110) in treatment-naive patients with acute myeloid leukaemia: Phase 2 results from a multicentre, randomised, phase 1/2 trial. Lancet Oncol..

[53] Businesswire. https://www.businesswire.com/news/home/20201014005914/en/.

[54] Savona M.R., Kolibaba K., Conkling P., Kingsley E.C., Becerra C., Morris J.C., Rifkin R.M., Laille E., Kellerman A., Ukrainskyj S.M. (2018). Extended dosing with CC-486 (oral azacitidine) in patients with myeloid malignancies. Am. J. Hematol..

[55] Garcia-Manero G., Scott B.L., Cogle C.R., Boyd T.E., Kambhampati S., Hetzer J., Dong Q., Kumar K., Ukrainskyj S.M., Beach C.L. (2018). CC-486 (oral azacitidine) in patients with myelodysplastic syndromes with pretreatment thrombocytopenia. Leuk Res..

[56] BMS. https://news.bms.com/news/details/2020/U.S.-Food-and-Drug-Administration-Approves-Onureg-azacitidine-tablets-a-New-Oral-Therapy-as-Continued-Treatment-for-Adults-in-First-Remission-with-Acute-Myeloid-Leukemia/default.aspx.

[57] Savona M.R., McCloskey J.K., Griffiths E.A., Yee K.W.L., Al-Kali A., Zeidan A.M., Deeg H.J., Patel P.A., Sabloff M., Keating M.-M. (2020). Clinical Efficacy and Safety of Oral Decitabine/Cedazuridine in 133 Patients with Myelodysplastic Syndromes (MDS) and Chronic Myelomonocytic Leukemia (CMML). Blood.

[58] Garcia-Manero G., McCloskey J., Griffiths E.A., Yee K.W.L., Zeidan A.M., Al-Kali A., Dao K.-H., Deeg H.J., Patel P.A., Sabloff M. (2019). Pharmacokinetic Exposure Equivalence and Preliminary Efficacy and Safety from a Randomized Cross over Phase 3 Study (ASCERTAIN study) of an Oral Hypomethylating Agent ASTX727 (cedazuridine/decitabine) Compared to IV Decitabine. Blood.

[59] FDA fda-approves-venetoclax-combination-aml-adults. https://www.fda.gov/drugs/fda-approves-venetoclax-combination-aml-adults.

[60] DiNardo C.D., Jonas B.A., Pullarkat V., Thirman M.J., Garcia J.S., Wei A.H., Konopleva M., Döhner H., Letai A., Fenaux P. (2020). Azacitidine and Venetoclax in Previously Untreated Acute Myeloid Leukemia. N. Engl. J. Med..

[61] Zeidan A.M., Pollyea D.A., Garcia J.S., Brunner A., Roncolato F., Borate U., Odenike O., Bajel A.R., Watson A.M., Götze K. (2019). A Phase 1b Study Evaluating the Safety and Efficacy of Venetoclax as Monotherapy or in Combination with Azacitidine for the Treatment of Relapsed/Refractory Myelodysplastic Syndrome. Blood.

[62] Medeiros B.C., Fathi A.T., DiNardo C.D., Pollyea D.A., Chan S.M., Swords R. (2017). Isocitrate dehydrogenase mutations in myeloid malignancies. Leukemia.

[63] Kennedy J.A., Ebert B.L. (2017). Clinical Implications of Genetic Mutations in Myelodysplastic Syndrome. J. Clin. Oncol..

[64] DiNardo C.D., Watts J.M., Stein E.M., de Botton S., Fathi A.T., Prince G.T., Stein A.S., Foran J.M., Stone R.M., Patel P.A. (2018). Ivosidenib (AG-120) Induced Durable Remissions and Transfusion Independence in Patients with IDH1-Mutant Relapsed or Refractory Myelodysplastic Syndrome: Results from a Phase 1 Dose Escalation and Expansion Study. Blood.

[65] Richard-Carpentier G., DeZern A.E., Takahashi K., Konopleva M.Y., Loghavi S., Masarova L., Alvarado Y., Ravandi F., Montalban Bravo G., Naqvi K. (2019). Preliminary Results from the Phase II Study of the IDH2-Inhibitor Enasidenib in Patients with High-Risk IDH2-Mutated Myelodysplastic Syndromes (MDS). Blood.

[66] Cortes J.E., Wang E.S., Watts J.M., Lee S., Baer M.R., Dao K.-H., Dinner S., Yang J., Donnellan W.B., Schwarer A.P. (2019). Olutasidenib (FT-2102) Induces Rapid Remissions in Patients with IDH1-Mutant Myelodysplastic Syndrome: Results of Phase 1/2 Single Agent Treatment and Combination with Azacitidine. Blood.

[67] Horiike S., Yokota S., Nakao M., Iwai T., Sasai Y., Kaneko H., Taniwaki M., Kashima K., Fujii H., Abe T. (1997). Tandem duplications of the FLT3 receptor gene are associated with leukemic transformation of myelodysplasia. Leukemia.

[68] Shih L.-Y., Huang C.-F., Wang P.-N., Wu J.-H., Lin T.-L., Dunn P., Kuo M.-C. (2004). Acquisition of FLT3 or N-ras mutations is frequently associated with progression of myelodysplastic syndrome to acute myeloid leukemia. Leukemia.

[69] Daver N., Strati P., Jabbour E., Kadia T., Luthra R., Wang S., Patel K., Ravandi F., Cortes J., Qin Dong X. (2013). *FLT3* mutations in myelodysplastic syndrome and chronic myelomonocytic leukemia. Am. J. Hematol..

[70] Badar T., Patel K.P., Thompson P.A., DiNardo C., Takahashi K., Cabrero M., Borthakur G., Cortes J., Konopleva M., Kadia T. (2015). Detectable FLT3-ITD or RAS mutation at the time of transformation from MDS to AML predicts for very poor outcomes. Leuk Res..

[71] Stone R.M., DeAngelo D.J., Klimek V., Galinsky I., Estey E., Nimer S.D., Grandin W., Lebwohl D., Wang Y., Cohen P. (2005). Patients with acute myeloid leukemia and an activating mutation in FLT3 respond to a small-molecule FLT3 tyrosine kinase inhibitor, PKC412. Blood.

[72] Fischer T., Stone R.M., Deangelo D.J., Galinsky I., Estey E., Lanza C., Fox E., Ehninger G., Feldman E.J., Schiller G.J. (2010). Phase IIB trial of oral Midostaurin (PKC412), the FMS-like tyrosine kinase 3 receptor (FLT3) and multi-targeted kinase inhibitor, in patients with acute myeloid leukemia and high-risk myelodysplastic syndrome with either wild-type or mutated FLT3. J. Clin. Oncol..

[73] Stone R.M., Mandrekar S.J., Sanford B.L., Laumann K., Geyer S., Bloomfield C.D., Thiede C., Prior T.W., Döhner K., Marcucci G. (2017). Midostaurin plus Chemotherapy for Acute Myeloid Leukemia with a *FLT3* Mutation. N. Engl. J. Med..

[74] Strati P., Kantarjian H., Ravandi F., Nazha A., Borthakur G., Daver N., Kadia T., Estrov Z., Garcia-Manero G., Konopleva M. (2015). Phase I/II trial of the combination of midostaurin (PKC412) and 5-azacytidine for patients with acute myeloid leukemia and myelodysplastic syndrome. Am. J. Hematol.

[75] FDA 021923_s000_NexavarTOC.cfm. https://www.accessdata.fda.gov/drugsatfda_docs/nda/2005/021923_s000_NexavarTOC.cfm.

[76] Burchert A., Bug G., Fritz L.V., Finke J., Stelljes M., Röllig C., Wollmer E., Wäsch R., Bornhäuser M., Berg T. (2020). Sorafenib Maintenance After Allogeneic Hematopoietic Stem Cell Transplantation for Acute Myeloid Leukemia With *FLT3* –Internal Tandem Duplication Mutation (SORMAIN). J. Clin. Oncol..

[77] Macdonald D.A., Assouline S.E., Brandwein J., Kamel-Reid S., Eisenhauer E.A., Couban S., Caplan S., Foo A., Walsh W., Leber B. (2013). A phase I/II study of sorafenib in combination with low dose cytarabine in elderly patients with acute myeloid leukemia or high-risk myelodysplastic syndrome from the National Cancer Institute of Canada Clinical Trials Group: Trial IND.186. Leuk Lymphoma.

[78] Ohanian M., Garcia-Manero G., Levis M., Jabbour E., Daver N., Borthakur G., Kadia T., Pierce S., Burger J., Richie M.A. (2018). Sorafenib Combined with 5-azacytidine in Older Patients with Untreated FLT3-ITD Mutated Acute Myeloid Leukemia. Am. J. Hematol..

[79] Park I.-K., Mishra A., Chandler J., Whitman S.P., Marcucci G., Caligiuri M.A. (2013). Inhibition of the receptor tyrosine kinase Axl impedes activation of the FLT3 internal tandem duplication in human acute myeloid leukemia: Implications for Axl as a potential therapeutic target. Blood.

[80] Park I.-K., Mundy-Bosse B., Whitman S.P., Zhang X., Warner S.L., Bearss D.J., Blum W., Marcucci G., Caligiuri M.A. (2015). Receptor tyrosine kinase Axl is required for resistance of leukemic cells to FLT3-targeted therapy in acute myeloid leukemia. Leukemia.

[81] FDA fda-approves-gilteritinib-relapsed-or-refractory-acute-myeloid-leukemia-aml-flt3-mutatation. https://www.fda.gov/drugs/fda-approves-gilteritinib-relapsed-or-refractory-acute-myeloid-leukemia-aml-flt3-mutatation.

[82] Cortes J.E., Kantarjian H., Foran J.M., Ghirdaladze D., Zodelava M., Borthakur G., Gammon G., Trone D., Armstrong R.C., James J. (2013). Phase I study of quizartinib administered daily to patients with relapsed or refractory acute myeloid leukemia irrespective of FMS-like tyrosine kinase 3-internal tandem duplication status. J. Clin. Oncol..

[83] Cortes J., Perl A.E., Döhner H., Kantarjian H., Martinelli G., Kovacsovics T., Rousselot P., Steffen B., Dombret H., Estey E. (2018). Quizartinib, an FLT3 inhibitor, as monotherapy in patients with relapsed or refractory acute myeloid leukaemia: An open-label, multicentre, single-arm, phase 2 trial. Lancet Oncol..

[84] Cortes J.E., Tallman M.S., Schiller G.J., Trone D., Gammon G., Goldberg S.L., Perl A.E., Marie J.-P., Martinelli G., Kantarjian H.M. (2018). Phase 2b study of 2 dosing regimens of quizartinib monotherapy in FLT3-ITD-mutated, relapsed or refractory AML. Blood.

[85] Visconte V., Nakashima O.M., Rogers J.H. (2019). Mutations in Splicing Factor Genes in Myeloid Malignancies: Significance and Impact on Clinical Features. Cancers.

[86] Maciejewski J.P., Padgett R.A. (2012). Defects in spliceosomal machinery: A new pathway of leukaemogenesis. Br. J. Haematol..

[87] Pellagatti A., Boultwood J. (2020). SF3B1 mutant myelodysplastic syndrome: Recent advances. Adv. Biol. Regul..

[88] Lee S.C.-W., Dvinge H., Kim E., Cho H., Micol J.-B., Chung Y.R., Durham B.H., Yoshimi A., Kim Y.J., Thomas M. (2016). Modulation of splicing catalysis for therapeutic targeting of leukemia with mutations in genes encoding spliceosomal proteins. Nat. Med..

[89] Seiler M., Yoshimi A., Darman R., Chan B., Keaney G., Thomas M., Agrawal A.A., Caleb B., Csibi A., Sean E. (2018). H3B-8800, an orally available small-molecule splicing modulator, induces lethality in spliceosome-mutant cancers. Nat. Med..

[90] Lee S.C.-W., Abdel-Wahab O. (2016). Therapeutic targeting of splicing in cancer. Nat. Med..

[91] Webb T.R., Joyner A.S., Potter P.M. (2013). The development and application of small molecule modulators of SF3b as therapeutic agents for cancer. Drug Discov. Today.

[92] Folco E.G., Coil K.E., Reed R. (2011). The anti-tumor drug E7107 reveals an essential role for SF3b in remodeling U2 snRNP to expose the branch point-binding region. Genes Dev..

[93] Han T., Goralski M., Gaskill N., Capota E., Kim J., Ting T.C., Xie Y., Williams N.S., Nijhawan D. (2017). Anticancer sulfonamides target splicing by inducing RBM39 degradation via recruitment to DCAF15. Science.

[94] Ungerstedt J. (2018). Epigenetic Modifiers in Myeloid Malignancies: The Role of Histone Deacetylase Inhibitors. Int. J. Mol. Sci..

[95] Goyama S., Kitamura T. (2017). Epigenetics in normal and malignant hematopoiesis: An overview and update 2017. Cancer Sci..

[96] Kim H.-J., Bae S.-C. (2011). Histone deacetylase inhibitors: Molecular mechanisms of action and clinical trials as anti-cancer drugs. Am. J. Transl. Res..

[97] San José-Enériz E., Gimenez-Camino N., Agirre X., Prosper F. (2019). HDAC Inhibitors in Acute Myeloid Leukemia. Cancers.

[98] Garcia-Manero G., Yang H., Bueso-Ramos C., Ferrajoli A., Cortes J., Wierda W.G., Faderl S., Koller C., Morris G., Rosner G. (2008). Phase 1 study of the histone deacetylase inhibitor vorinostat (suberoylanilide hydroxamic acid [SAHA]) in patients with advanced leukemias and myelodysplastic syndromes. Blood.

[99] Kuendgen A., Schmid M., Schlenk R., Knipp S., Hildebrandt B., Steidl C., Germing U., Haas R., Dohner H., Gattermann N. (2006). The histone deacetylase (HDAC) inhibitor valproic acid as monotherapy or in combination with all-trans retinoic acid in patients with acute myeloid leukemia. Cancer.

[100] O’Connor O.A., Heaney M.L., Schwartz L., Richardson S., Willim R., MacGregor-Cortelli B., Curly T., Moskowitz C., Portlock C., Horwitz S. (2006). Clinical experience with intravenous and oral formulations of the novel histone deacetylase inhibitor suberoylanilide hydroxamic acid in patients with advanced hematologic malignancies. J. Clin. Oncol..

[101] Gojo I., Jiemjit A., Trepel J.B., Sparreboom A., Figg W.D., Rollins S., Tidwell M.L., Greer J., Chung E.J., Lee M.-J. (2007). Phase 1 and pharmacologic study of MS-275, a histone deacetylase inhibitor, in adults with refractory and relapsed acute leukemias. Blood.

[102] Garcia-Manero G., Tambaro F.P., Bekele N.B., Yang H., Ravandi F., Jabbour E., Borthakur G., Kadia T.M., Konopleva M.Y., Faderl S. (2012). Phase II trial of vorinostat with idarubicin and cytarabine for patients with newly diagnosed acute myelogenous leukemia or myelodysplastic syndrome. J. Clin. Oncol..

[103] Garcia-Manero G., Sekeres M.A., Egyed M., Breccia M., Graux C., Cavenagh J.D., Salman H., Illes A., Fenaux P., DeAngelo D.J. (2017). A phase 1b/2b multicenter study of oral panobinostat plus azacitidine in adults with MDS, CMML or AML with ≤30% blasts. Leukemia.

[104] Sekeres M.A., Othus M., List A.F., Odenike O., Stone R.M., Gore S.D., Litzow M.R., Buckstein R., Fang M., Roulston D. (2017). Randomized Phase II Study of Azacitidine Alone or in Combination With Lenalidomide or With Vorinostat in Higher-Risk Myelodysplastic Syndromes and Chronic Myelomonocytic Leukemia: North American Intergroup Study SWOG S1117. J. Clin. Oncol..

[105] Garcia-Manero G., Montalban-Bravo G., Berdeja J.G., Abaza Y., Jabbour E., Essell J., Lyons R.M., Ravandi F., Maris M., Heller B. (2017). Phase 2, randomized, double-blind study of pracinostat in combination with azacitidine in patients with untreated, higher-risk myelodysplastic syndromes. Cancer.

[106] Prebet T., Sun Z., Ketterling R.P., Zeidan A., Greenberg P., Herman J., Juckett M., Smith M.R., Malick L., Paietta E. (2016). Azacitidine with or without Entinostat for the treatment of therapy-related myeloid neoplasm: Further results of the E1905 North American Leukemia Intergroup study. Br. J. Haematol..

[107] Maslah N., Salomao N., Drevon L., Verger E., Partouche N., Ly P., Aubin P., Naoui N., Schlageter M.-H., Bally C. (2020). Synergistic effects of PRIMA-1Met (APR-246) and 5-azacitidine in TP53-mutated myelodysplastic syndromes and acute myeloid leukemia. Haematologica.

[108] Sallman D.A., DeZern A.E., Garcia-Manero G., Steensma D.P., Roboz G.J., Sekeres M.A., Cluzeau T., Sweet K.L., McLemore A.F., McGraw K. (2019). Phase 2 Results of APR-246 and Azacitidine (AZA) in Patients with TP53 mutant Myelodysplastic Syndromes (MDS) and Oligoblastic Acute Myeloid Leukemia (AML). Blood.

[109] Cluzeau T., Sebert M., Rahmé R., Cuzzubbo S., Walter-petrich A., Lehmann che J., Peterlin P., Beve B., Attalah H., Chermat F. (2019). APR-246 Combined with Azacitidine (AZA) in TP53 Mutated Myelodysplastic Syndrome (MDS) and Acute Myeloid Leukemia (AML). a Phase 2 Study By the Groupe Francophone Des Myélodysplasies (GFM). Blood.

[110] Abou Zahr A., Saad Aldin E., Komrokji R.S., Zeidan A.M. (2015). Clinical utility of lenalidomide in the treatment of myelodysplastic syndromes. J. Blood Med..

[111] Fink E.C., Ebert B.L. (2015). The novel mechanism of lenalidomide activity. Blood.

[112] Krönke J., Fink E.C., Hollenbach P.W., MacBeth K.J., Hurst S.N., Udeshi N.D., Chamberlain P.P., Mani D.R., Man H.W., Gandhi A.K. (2015). Lenalidomide induces ubiquitination and degradation of CK1α in del(5q) MDS. Nature.

[113] Giagounidis A., Mufti G.J., Fenaux P., Germing U., List A., MacBeth K.J. (2014). Lenalidomide as a disease-modifying agent in patients with del(5q) myelodysplastic syndromes: Linking mechanism of action to clinical outcomes. Ann. Hematol..

[114] Fenaux P., Giagounidis A., Selleslag D., Beyne-Rauzy O., Mufti G., Mittelman M., Muus P., Te Boekhorst P., Sanz G., del Cañizo C. (2011). A randomized phase 3 study of lenalidomide versus placebo in RBC transfusion-dependent patients with Low-/Intermediate-1-risk myelodysplastic syndromes with del5q. Blood.

[115] List A., Dewald G., Bennett J., Giagounidis A., Raza A., Feldman E., Powell B., Greenberg P., Thomas D., Stone R. (2006). Lenalidomide in the Myelodysplastic Syndrome with Chromosome 5q Deletion. N. Engl. J. Med..

[116] Oliva E.N., Latagliata R., Laganà C., Breccia M., Galimberti S., Morabito F., Poloni A., Balleari E., Cortelezzi A., Palumbo G. (2013). Lenalidomide in International Prognostic Scoring System Low and Intermediate-1 risk myelodysplastic syndromes with del(5q): An Italian phase II trial of health-related quality of life, safety and efficacy. Leuk. Lymphoma.

[117] Santini V., Almeida A., Giagounidis A., Gröpper S., Jonasova A., Vey N., Mufti G.J., Buckstein R., Mittelman M., Platzbecker U. (2016). Randomized Phase III Study of Lenalidomide Versus Placebo in RBC Transfusion-Dependent Patients With Lower-Risk Non-del(5q) Myelodysplastic Syndromes and Ineligible for or Refractory to Erythropoiesis-Stimulating Agents. J. Clin. Oncol..

[118] Adès L., Boehrer S., Prebet T., Beyne-Rauzy O., Legros L., Ravoet C., Dreyfus F., Stamatoullas A., Chaury M.P., Delaunay J. (2009). Efficacy and safety of lenalidomide in intermediate-2 or high-risk myelodysplastic syndromes with 5q deletion: Results of a phase 2 study. Blood.

[119] Möllgård L., Saft L., Treppendahl M.B., Dybedal I., Nørgaard J.M., Astermark J., Ejerblad E., Garelius H., Dufva I.H., Jansson M. (2011). Clinical effect of increasing doses of lenalidomide in high-risk myelodysplastic syndrome and acute myeloid leukemia with chromosome 5 abnormalities. Haematologica.

[120] Cherian M.A., Tibes R., Gao F., Fletcher T., Fiala M., Uy G.L., Westervelt P., Jacoby M.A., Cashen A.F., Stockerl-Goldstein K. (2016). A study of high-dose lenalidomide induction and low-dose lenalidomide maintenance therapy for patients with hypomethylating agent refractory myelodysplastic syndrome. Leuk Lymphoma.

[121] Sekeres M.A., Tiu R.V., Komrokji R., Lancet J., Advani A.S., Afable M., Englehaupt R., Juersivich J., Cuthbertson D., Paleveda J. (2012). Phase 2 study of the lenalidomide and azacitidine combination in patients with higher-risk myelodysplastic syndromes. Blood.

[122] Winter S., Shoaie S., Kordasti S., Platzbecker U. (2020). Integrating the “Immunome” in the Stratification of Myelodysplastic Syndromes and Future Clinical Trial Design. J. Clin. Oncol..

[123] Masarova L., Kantarjian H., Ravandi F., Sharma P., Garcia-Manero G., Daver N. (2018). Update on Immunotherapy in AML and MDS: Monoclonal Antibodies and Checkpoint Inhibitors Paving the Road for Clinical Practice. Adv. Exp. Med. Biol..

[124] Chokr N., Patel R., Wattamwar K., Chokr S. (2018). The Rising Era of Immune Checkpoint Inhibitors in Myelodysplastic Syndromes. Adv. Hematol..

[125] Zeidan A.M., Knaus H.A., Robinson T.M., Towlerton A.M.H., Warren E.H., Zeidner J.F., Blackford A.L., Duffield A.S., Rizzieri D., Frattini M.G. (2018). A Multi-center Phase I Trial of Ipilimumab in Patients with Myelodysplastic Syndromes following Hypomethylating Agent Failure. Clin. Cancer Res..

[126] Garcia-Manero G., Tallman M.S., Martinelli G., Ribrag V., Yang H., Balakumaran A., Chlosta S., Zhang Y., Smith B.D. (2016). Pembrolizumab, a PD-1 Inhibitor, in Patients with Myelodysplastic Syndrome (MDS) after Failure of Hypomethylating Agent Treatment. Blood.

[127] Garcia-Manero G., Sasaki K., Montalban-Bravo G., Daver N.G., Jabbour E.J., Alvarado Y., DiNardo C.D., Ravandi F., Borthakur G., Bose P. (2018). A Phase II Study of Nivolumab or Ipilimumab with or without Azacitidine for Patients with Myelodysplastic Syndrome (MDS). Blood.

[128] Zeidan A.M., Cavenagh J., Voso M.T., Taussig D., Tormo M., Boss I., Copeland W.B., Gray V.E., Previtali A., O’Connor T. (2019). Efficacy and Safety of Azacitidine (AZA) in Combination with the Anti-PD-L1 Durvalumab (durva) for the Front-Line Treatment of Older Patients (pts) with Acute Myeloid Leukemia (AML) Who Are Unfit for Intensive Chemotherapy (IC) and Pts with Higher-Risk Myelodysplastic Syndromes (HR-MDS): Results from a Large, International, Randomized Phase 2 Study. Blood.

[129] Ozkazanc D., Yoyen-Ermis D., Tavukcuoglu E., Buyukasik Y., Esendagli G. (2016). Functional exhaustion of CD4+ T cells induced by co-stimulatory signals from myeloid leukaemia cells. Immunology.

[130] Anderson A.C. (2014). Tim-3: An Emerging Target in the Cancer Immunotherapy Landscape. Cancer Immunol. Res..

[131] Asayama T., Tamura H., Ishibashi M., Kuribayashi-Hamada Y., Onodera-Kondo A., Okuyama N., Yamada A., Shimizu M., Moriya K., Takahashi H. (2017). Functional expression of Tim-3 on blasts and clinical impact of its ligand galectin-9 in myelodysplastic syndromes. Oncotarget.

[132] Borate U., Esteve J., Porkka K., Knapper S., Vey N., Scholl S., Garcia-Manero G., Wermke M., Janssen J., Traer E. (2019). Phase Ib Study of the Anti-TIM-3 Antibody MBG453 in Combination with Decitabine in Patients with High-Risk Myelodysplastic Syndrome (MDS) and Acute Myeloid Leukemia (AML). Blood.

[133] Hatherley D., Graham S.C., Turner J., Harlos K., Stuart D.I., Barclay A.N. (2008). Paired receptor specificity explained by structures of signal regulatory proteins alone and complexed with CD47. Mol. Cell.

[134] Iribarren K., Buque A., Mondragon L., Xie W., Lévesque S., Pol J., Zitvogel L., Kepp O., Kroemer G. (2019). Anticancer effects of anti-CD47 immunotherapy in vivo. Oncoimmunology.

[135] Folkes A.S., Feng M., Zain J.M., Abdulla F., Rosen S.T., Querfeld C. (2018). Targeting CD47 as a cancer therapeutic strategy: The cutaneous T-cell lymphoma experience. Curr. Opin. Oncol..

[136] Sallman D.A., Asch A.S., Al Malki M.M., Lee D.J., Donnellan W.B., Marcucci G., Kambhampati S., Daver N.G., Garcia-Manero G., Komrokji R.S. (2019). The First-in-Class Anti-CD47 Antibody Magrolimab (5F9) in Combination with Azacitidine Is Effective in MDS and AML Patients: Ongoing Phase 1b Results. Blood.

[137] Massagué J. (2008). TGFβ in Cancer. Cell.

[138] Bewersdorf J.P., Zeidan A.M. (2019). Transforming growth factor (TGF)-β pathway as a therapeutic target in lower risk myelodysplastic syndromes. Leukemia.

[139] Platzbecker U., Germing U., Götze K.S., Kiewe P., Mayer K., Chromik J., Radsak M., Wolff T., Zhang X., Laadem A. (2017). Luspatercept for the treatment of anaemia in patients with lower-risk myelodysplastic syndromes (PACE-MDS): A multicentre, open-label phase 2 dose-finding study with long-term extension study. Lancet Oncol..

[140] Komrokji R., Garcia-Manero G., Ades L., Prebet T., Steensma D.P., Jurcic J.G., Sekeres M.A., Berdeja J., Savona M.R., Beyne-Rauzy O. (2018). Sotatercept with long-term extension for the treatment of anaemia in patients with lower-risk myelodysplastic syndromes: A phase 2, dose-ranging trial. Lancet Haematol..

[141] Fenaux P., Platzbecker U., Mufti G.J., Garcia-Manero G., Buckstein R., Santini V., Díez-Campelo M., Finelli C., Cazzola M., Ilhan O. (2020). Luspatercept in Patients with Lower-Risk Myelodysplastic Syndromes. N. Engl. J. Med..

[142] FDA fda-approves-luspatercept-aamt-anemia-adults-mds. https://www.fda.gov/drugs/resources-information-approved-drugs/fda-approves-luspatercept-aamt-anemia-adults-mds.

[143] Athuluri-Divakar S.K., Vasquez-Del Carpio R., Dutta K., Baker S.J., Cosenza S.C., Basu I., Gupta Y.K., Reddy M.V.R., Ueno L., Hart J.R. (2016). A Small Molecule RAS-Mimetic Disrupts RAS Association with Effector Proteins to Block Signaling. Cell.

[144] Komrokji R.S., Raza A., Lancet J.E., Ren C., Taft D., Maniar M., Wilhelm F., List A.F. (2013). Phase I clinical trial of oral rigosertib in patients with myelodysplastic syndromes. Br. J. Haematol..

[145] Navada S.C., Fruchtman S.M., Odchimar-Reissig R., Demakos E.P., Petrone M.E., Zbyszewski P.S., Holland J.F., Silverman L.R. (2018). A phase 1/2 study of rigosertib in patients with myelodysplastic syndromes (MDS) and MDS progressed to acute myeloid leukemia. Leuk. Res..

[146] Silverman L.R., Greenberg P., Raza A., Olnes M.J., Holland J.F., Reddy P., Maniar M., Wilhelm F. (2015). Clinical activity and safety of the dual pathway inhibitor rigosertib for higher risk myelodysplastic syndromes following DNA methyltransferase inhibitor therapy. Hematol. Oncol..

[147] Garcia-Manero G., Fenaux P., Al-Kali A., Baer M.R., Sekeres M.A., Roboz G.J., Gaidano G., Scott B.L., Greenberg P., Platzbecker U. (2016). Rigosertib versus best supportive care for patients with high-risk myelodysplastic syndromes after failure of hypomethylating drugs (ONTIME): A randomised, controlled, phase 3 trial. Lancet Oncol..

[148] Garcia-Manero G., Navada S.C., Fenaux P., Zbyszewski P.S., Adesanya A.R., Azarnia N., Fruchtman S.M., Silverman L.R. (2019). Phase 3, Multi-Center, International, Randomized, Double-Blind, Placebo Controlled Study of Oral Rigosertib + Injectable Azacitidine (AZA) Versus Injectable Azacitidine in Treatment-Naive Patients with Higher-Risk Myelodysplastic Syndrome (HR-MDS). Blood.

[149] Cortes J.E., Douglas Smith B., Wang E.S., Merchant A., Oehler V.G., Arellano M., DeAngelo D.J., Pollyea D.A., Sekeres M.A., Robak T. (2018). Glasdegib in combination with cytarabine and daunorubicin in patients with AML or high-risk MDS: Phase 2 study results. Am. J. Hematol..

[150] Cortes J.E., Heidel F.H., Hellmann A., Fiedler W., Smith B.D., Robak T., Montesinos P., Pollyea D.A., DesJardins P., Ottmann O. (2019). Randomized comparison of low dose cytarabine with or without glasdegib in patients with newly diagnosed acute myeloid leukemia or high-risk myelodysplastic syndrome. Leukemia.

[151] Sallman D.A., Komrokji R.S., Sweet K.L., Mo Q., McGraw K.L., Duong V.H., Zhang L., Nardelli L.A., Padron E., List A.F. (2019). A phase 2 trial of the oral smoothened inhibitor glasdegib in refractory myelodysplastic syndromes (MDS). Leuk Res..

[152] Swords R.T., Coutre S., Maris M.B., Zeidner J.F., Foran J.M., Cruz J., Erba H.P., Berdeja J.G., Tam W., Vardhanabhuti S. (2018). Pevonedistat, a first-in-class NEDD8-activating enzyme inhibitor, combined with azacitidine in patients with AML. Blood.

[153] Moyo T.K., Watts J.M., Skikne B.S., Mendler J.H., Klimek V.M., Chen S.-C., Fan R., Anderson I.A., Sochacki A., Strickland S.A. (2019). Preliminary Results from a Phase II Study of the Combination of Pevonedistat and Azacitidine in the Treatment of MDS and MDS/MPN after Failure of DNA Methyltransferase Inhibition. Blood.

[154] Ades L., Watts J.M., Radinoff A., Arnan M., Cerrano M., Font Lopez P., Zeidner J.F., Diez-Campelo M., Graux C., Liesveld J. (2020). Phase II study of pevonedistat (P) + azacitidine (A) versus A in patients (pts) with higher-risk myelodysplastic syndromes (MDS)/chronic myelomonocytic leukemia (CMML), or low-blast acute myelogenous leukemia (LB AML) (NCT02610777). J. Clin. Oncol..

[155] Chen N., Hao C., Liu B.-C., Lin H., Wang C., Xing C., Liang X., Jiang G., Liu Z., Li X. (2019). Roxadustat Treatment for Anemia in Patients Undergoing Long-Term Dialysis. N. Engl. J. Med..

[156] Dhillon S. (2019). Roxadustat: First Global Approval. Drugs.

[157] Wu L., Fidan K., Um J.-Y., Ahn K.S. (2020). Telomerase: Key regulator of inflammation and cancer. Pharmacol. Res..

[158] Steensma D.P., Fenaux P., Van Eygen K., Raza A., Santini V., Germing U., Font P., Diez-Campelo M., Thepot S., Vellenga E. (2021). Imetelstat Achieves Meaningful and Durable Transfusion Independence in High Transfusion–Burden Patients With Lower-Risk Myelodysplastic Syndromes in a Phase II Study. J. Clin. Oncol..

[159] Sanford D., Lo-Coco F., Sanz M.A., Di Bona E., Coutre S., Altman J.K., Wetzler M., Allen S.L., Ravandi F., Kantarjian H. (2015). Tamibarotene in patients with acute promyelocytic leukaemia relapsing after treatment with all-trans retinoic acid and arsenic trioxide. Br. J. Haematol..

[160] De Botton S., Cluzeau T., Vigil C.E., Cook R.J., Rousselot P., Rizzieri D.A., Liesveld J.L., Fenaux P., Braun T., Banos A. (2020). SY-1425, a Potent and Selective RARα Agonist, in Combination with Azacitidine Demonstrates a High Complete Response Rate and a Rapid Onset of Response in RARA-Positive Newly Diagnosed Unfit Acute Myeloid Leukemia. Blood.

